# Narrative constructs in modern clinical case reporting

**DOI:** 10.1016/j.shpsa.2017.03.004

**Published:** 2017-04

**Authors:** Brian Hurwitz

**Affiliations:** King's College London, London, WC2B 6LE, United Kingdom

**Keywords:** Narrative, Case, Case history, Case report, Disease, Illness

## Abstract

Modern clinical case reporting takes the form of problem-solution narratives that redescribe symptoms in terms of disease categories. Authored almost always by those who have played a part in the medical assessment of the patient, reports historicise the salient details of an individual's illness as a complex effect of identifiable antecedent causes. Candidate hypotheses linking illness to pathological mechanisms are suggested by the patient’s experience, and by data that emerge from clinical examination and investigation. Observational and interpretive statements from these considerations are fitted into a temporally inflected account of the patient’s medical condition, configured from the vantage point of hindsight. Drawing on established forms of deferred telling, readers are invited to follow a story that drip-feeds a mixture of contingent and non-incidental information into the account, which engenders and frustrates curiosity, creates expectations, and challenges powers of reasoning and pattern recognition. Whereas case reporting once favoured memoir, the sentimental tale and eccentric biography as the means by which its historical narrative was cast, the preferred genres of contemporary case reporting include detective fiction, and puzzle and riddle narratives, formats that conceptualise the medical consultation in narrow problem-solution terms.

## 1 Introduction: not if p but which p?

Modern clinical case reports are problem-solution accounts of how an individual's felt experiences of illness have come to be understood in terms of disease categories. Authored almost invariably by those who have played some part in the medical assessment of the patient, published case reports are crafted statements of witness marked by scene-setting strategies and graphic descriptions of clinical findings. What John Forrester verbalized as ‘thinking in cases,’^1^ contemporary clinical case reports recount through controlled disclosures of observations and reasoning, positioned with the benefit of hindsight in a narrative.

Forrester's paper “If *p*, then what?” is credited with refocusing interest on case construals as pathways to understanding the methods and reasonings of case-based disciplines.^2^ Whilst his paper focused largely on the ‘then what’ that follows from *p* – where *p* for the most part is unproblematically known - clinical cases devote greater attention to observations and the possible multiplicity of *p*; to what deserves to be noticed in view of the need to differentiate *p*_1_-*p*_n_, and so to fathom which *p* fits the case in hand.

*The Lancet* recently announced it had increased the space devoted to publishing case reports, particularly those elucidating ‘an unusual presentation of a common disease or a rare cause of a common presentation if not something completely novel.’^3^ The editors contended that ‘stories form the basis of how we learn, and how we remember’ and that case reporting practices embody clinical experiences that collectively constitute a written repository comparable to other literary and cultural genres:

‘Throughout history people have interpreted the world around them and passed on lessons learned through myths, fairy tales, parables, and anecdotes. Medicine is no different … The ideal Case Report will have an unexpected twist or detective element, is engagingly written, and has a learning point for a general medical audience.’^4^

Twenty years earlier the journal had established a new section on case reporting to enable clinicians to ‘relay the sort of clinical anecdote they might tell colleagues during a morning coffee break’ in despatches from the clinical frontline conveying ‘a striking message: a description of a new treatment, adverse effect of medication, evidence that might suggest a new mechanism for a disease process, or a new intervention.’^5^ That initiative marked the point when the post WWII decline in medical publication of single cases was coming to an end. Despite their uncontrolled, non-experimental nature, likelihood of observer bias and inherent inability to estimate the prevalence or incidence of clinical features of interest, by the end of the twentieth century case reports were increasingly recognized as playing important if not unique evidential roles in medical practice. These roles included bringing to light very rare clinical phenomena, delineating initial descriptions of previously unrecognised diseases such as HIV, Ebola and Lyme disease,^6^ demonstrating the concurrence of clinical symptoms and signs constellated as syndromes,^7^ and in identifying and defining adverse drug reactions.^8^ The importance of these roles re-instilled interest in clinical cases and led to what has been called a ‘renaissance of the case report literature’.^9^ This paper will draw out the narrative scaffoldings of contemporary medical case reports, their interplay with other storied genres, and how clinical findings and their explanations become enmeshed in the literary machinery of reporting.

Forrester acknowledged a pedagogic aspect to cases that ‘duplicates or repeats an essential element of medical practice’,^10^ in a form of writing ‘epistemically … nailed down to the level of the individual’.^11^ But although the case report ‘brings back’^12^ elements of the clinical encounter arising from and pertaining to a particular individual, its ostensive focus is on the medical condition, syndrome, or treatment and its effects, not the person who is ill. However, tension between these potential foci of case reporting practices will become apparent. Consider a clinical vignette that appeared in the *British Medical Journal* in 2007 in a paper entitled “When are randomised trials unnecessary?” to exhibit the authors' claim that ‘the relation between a treatment and its effect is sometimes so dramatic that bias can be ruled out as an explanation’:

‘A child presented to a clinic with a plastic bead lodged high in one nostril. The general practitioner asked the nurse for forceps, but she asked him whether he had thought of trying the mother's kiss technique. This entailed occluding the unblocked nostril while the mother blew into the child's mouth. The bead was easily dislodged and retrieved in this way, and mother and child were both delighted.’^13^

In sketching a manoeuvre to relieve this relatively common childhood condition the authors convey something of the atmosphere of the clinic, its voices, emotions and sounds – dialogue and ‘delight’ – which serves to convince readers that the account is grounded in the realities of daily clinical work. The vignette signals clinical verisimilitude and an immediate therapeutic effect, an instance of a generalization applicable beyond the singularities of this particular child.

Elements of the scenario are plainly incidental to the causal claim of interest, such as the conversation between doctor and nurse (a nurse who appears to be better informed than the doctor), which steers treatment away from a more traumatic extraction with forceps, to a focus on mother and child and on a cooperative procedure. Such details can be read as valorising clinical teamwork and the doctor's willingness to try a treatment he or she has not previously thought of. But these aspects of the account also point to a degree of contingency in the situation – what would have happened had a different nurse been on duty or no nurse at all? – and raise the possibility that a procedure that could have been instituted was not, a counterfactual which endows the scene with social and human significance, whilst dramatizing the cause and effect sequence on display (see Beatty this issue).^14^

Viewed in isolation, the vignette recounts an observation with a persuasive power untempered by considerations such as: snugness of fit between the bead and nasal lining; how long it has been in the child's nose; whether prior attempts at retrieval have pushed the bead upwards; whether there is a purulent nasal discharge (a sign of mucosal ulceration and secondary infection); and the sort of blowing required to dislodge the bead, be it sharp bursts or the creation of a continuously rising pressure wave.^15^ In paying no heed to these factors - to which particular *p* this child's situation belongs - which has implications for the relevant treatment^16^ - the vignette outlines an almost paradigmatic instance of the manoeuvre's efficacy,^17^ its persuasiveness bolstered by the ebullient mood and celebratory tone of the narrative's affecting denouement.

However, some of the details of the emplotment here may not be as incidental to the efficacy of the procedure as they may appear, such as the plastic nature of the misplaced bead - if this implies it was round and smooth that may account for the ‘easy’ removal - and the fact that success transpired on the first attempt. The significance of such details are suggested by other case reports which consider factors on which success may hinge such as the nature, size, shape and regularity of the object in the nasal cavity and how many ‘kisses’ are worth attempting.^18^ Success may also depend on the way a young child is approached to win their cooperation, as not all blowers are likely to gain the cooperative involvement that is required.^19^ Which descriptive details are incidental and which are material to the efficacy of the manoeuvre reflects the heterogeneity of misplaced objects in the nasal cavity, the age and cooperativeness of children with this problem: the diversity in *p*.

Although the actors in this situation are anonymous and characterized in only the sketchiest of terms, the vignette invokes ‘particular individuals living particular lives and having particular experiences’^20^ as well as a determination to understand their situation ‘from the perspective of the general’.^21^ Steve Sturdy argues that in relation to ‘knowledge of cases the content or meaning of any scientific generalization is no more than the finite set of cases that are taken to be instances or exemplifications of that generalization.’^22^ The sparsity of the vignette's details appears to exemplify a generalization of wide scope unqualified by the differing circumstances of children with a misplaced foreign body in their nostrils.

Despite its brevity the vignette ties together four individuals in a pairwise cooperative venture that shifts attention away from the ostensive medical focus on a cause and effect relationship of clear therapeutic benefit to the human relationships involved in the clinical scenario. It thereby illustrates George Rousseau's claim that ‘every time a patient enters a practitioner's office a literary experience is about to occur: replete with characters, setting, time, place, language and a scenario that can end in a number of predictable ways.’^23^

## 2 The narrative: detective novels and the gothic case

In setting out how illness or injury can be translated into a diagnosis, case reporting attempts to match selected elements of illness experience and clinical findings with plausible medical causes and mechanisms. Confronted with ‘the patient's messy humanity’,^24^ Jason Tougaw argues that the narrator picks out and orders elements abstracted from the history and examination of the patient (see Morgan this issue), in an ‘attempt to make signs and things match’, contemporary reporting practices favoring certain recognisable genres. One narrative template has gained particular prominence notes Kathryn Hunter:

‘Like a Sherlock Holmes story, the case presentation is … concerned with the way knowledge is acquired as well as with the nature of the “facts”… Like the criminal… the disease in the case presentation is discovered in a double sense: it is determined by the investigating physician and revealed by the same person, now become the narrator … discovery proceeds from the careful arrangement of data which the physician… has gathered from the words and the body presented for scrutiny.’^25^

Detective and clinical case work share imaginative, interrogative and inferential moves inflected in appeals to puzzle out and fit together diverse pieces of information in ‘a single connected narrative’.^26^ In both fields of activity accounts of cases defer telling by withholding information and concealing elements as in a ‘game of suspense and mystery’.^27^ The emergence of pattern is orchestrated through a process that stages access to findings by drip-feeding descriptive information in which reasoning and the formation of hypotheses are the intermediary links of narrative beginnings and endings (see Rosales's discussion in this issue). Lucy Sussex finds the puzzle-making and unriddling activities of nineteenth-century detective fiction^28^ to have been informed by a gothic aesthetic derived from suspenseful and sensational plots. David Punter picks up on this in his study of *Gothic Pathologies*:

‘The case is the compound of rule and singularity. When we talk of ‘case law’ we talk of the importance of the individual case… the exception which proves the rule, or …the rule which brooks no exception…. The case is also the suitcase, the briefcase, the container… What Gothic shows is that this case always falls open (and, as it were, things fall out).’^29^

Drawing on the semantic burden case inherits from the Old French word casse, meaning box or frame, shell or carcass, Punter stresses the importance of suspense and surprise in the construction of fictional cases, and hints at how access to secrets and narrative mystery engenders not only curiosity but guessing and conjecturing too. For Carole Levine these devices play an epistemological role: ‘as we read suspenseful plots we learn to doubt and to guess, to speculate and hypothesise, to pause in the knowledge of what we do *not* know’.^30^ Suspenseful plots provide a training ground for learning to re-consider what within an account has gone before, before jumping to conclusions. ‘Suspense demands a … quite specific experience of the unknown’, she says, an awareness of ignorance:

‘Even when the solution to the mystery ultimately substantiates our hopes and predictions, for there to have been suspense - for us to have remained absorbed, apprehensive, doubtful … we must have been willing to entertain a range of credible resolutions.’^31^

The following case, which appeared in the *British Medical Journal* in 1995, drives home its lesson by ostentatiously playing with uncertainty and anticipation:

‘A builder aged 29 came to the accident and emergency department having jumped down on to a 15 cm nail. As the smallest movement of the nail was painful he was sedated with fentanyl and midazolam. The nail was then pulled out from below. When his boot was removed a miraculous cure appeared to have taken place. Despite entering proximal to the steel toecap the nail had penetrated between the toes: the foot was entirely uninjured.’^32^

**Fig. 1:**
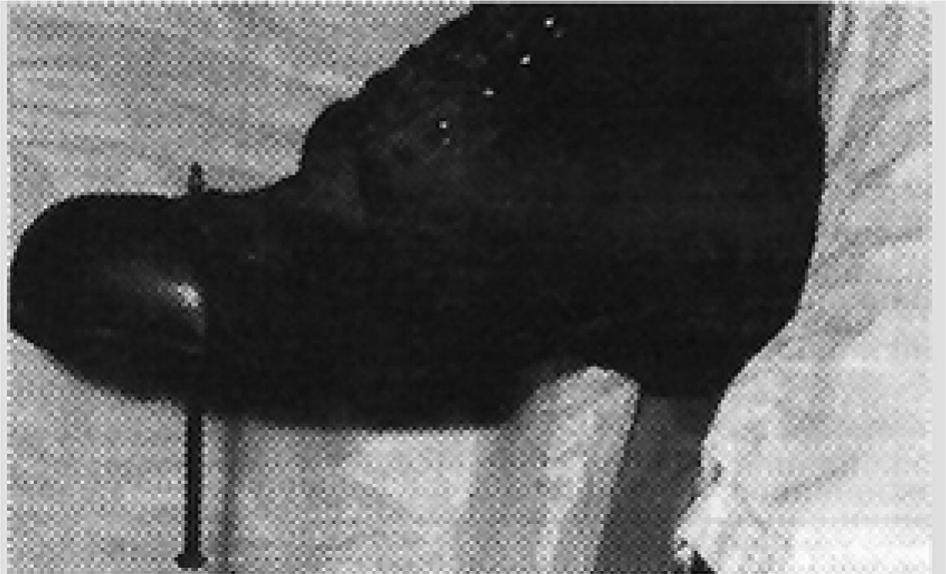
Reproduced from the *British Medical Journal,* Fisher JP, Hassan DT, O'Connor N., Vol 310 page 70, (copyright notice 4003140950535 2016) with permission from BMJ Publishing Group Ltd.

The account unfolds along an apparently straightforward timeline, recounting an injury causing pain severe enough to require a potent opioid analgesic before nail extraction is attempted. However, when readers textually and visually primed for an impalement injury are advised to expect a ‘cure,’ the narrative flirts with expectation and assumes the characteristics of a riddle. The clinician-authors play a mimetic trick on readers who are prepared for a nasty wound but instead experience the same sort of surprise the authors must have felt on removing the man's boot (see Fig. 1).

The promise of a ‘miraculous cure’ turns out to be a false trail laid to inflict momentary astonishment and bewilderment on readers. The report duplicates something of the consternation felt in the Emergency department and reveals that the urge to conjure up the enigmatic - to entertain and shock with spectacle in what Rick Rylance refers to as ‘shifts between gothic make-believe and the modernized medical mode’^33^ – is still at work in case reporting today.

But the nail's innocent anatomical position raises an additional important question omitted from consideration: why did the man feel so much pain? The report also leaves readers to ponder how the workman reacted when he realized he was unharmed: was he relieved, did he laugh at the joke his body had played on him and on his medical attendants; or was he mystified by this turn of events? Other contextual questions are left dangling such as: what sort of a person the uninjured man was; was he an anxious, frightened, unconfident individual; did he have a phobia of hospitals; had he previously suffered serious painful accidents; is he the sort of person who ‘acts out’ when feeling threatened?^34^ Questions such as these show how unsatisfying case closure can be, and how much relevant information about the person from whom the case arises may be excluded from a case report; questions that challenge the separation of person (with a psychology) from the medical condition under scrutiny, which conventions of case reporting impose. The distinction between the person who is ill (the patient), and the abstract category from which they suffer (the disease or medical condition), appears porous if not unstable.^35^

## 3 Taking the patient's history; telling the doctor's story

Julia Epstein argues that ‘Western medical discourse postulates that illness can be, at least momentarily (long enough… to study, classify, and pass judgment on it), dissociable from the ill person.’^36^ In case reporting, as we have seen, this dissociation shows signs of instability. Consider a case reported in the *British Medical Journal* in 2010 under the running title of “Endgames”:

‘A 25 year old woman presented to our institution with increasing difficulty in swallowing, talking, and walking, which resulted in her being virtually mute, wheelchair bound, and perpetually drooling. She had presented to another institution two and a half years before with worsening episodes of headache and cramp in both feet. This had progressed to a generalised weakness of her entire body, in addition to impaired speech and problems with motor coordination. Clinical examination showed cog wheel rigidity and a generalised jerky dystonic tremor, with occasional hemiballismic movement of the arms … brisk reflexes and severe spasticity of the lower limbs. Her speech was slurred and bilateral corneal discoloration was noted.’^37^

The diagnoses considered – in the process of deciding which *p* is the relevant one - include a variant of Parkinson's disease; pantothenate kinase associated neurodegeneration (a condition in which iron accumulates and damages the central nervous system); and Wilson's disease: a genetic disorder of copper metabolism in which the copper binding protein ceruloplasmin, that transports the element within the body, is almost completely absent, leading to the toxic element accumulating, and causing tissue damage in the nervous system. Shortly after its publication the following letter appeared in the journal:

‘Why on earth was a (presumably) previously healthy 25yo woman allowed to degenerate to the point of being wheelchair-bound, mute, and drooling without apparently having been seen by a single physician who would not rest until a diagnosis has been made? Not wishing to cast aspersions, but there is something very wrong here. Was she a refugee from a country without even a semblance of adequate health care, or was her family so passive as to just accept this awful decline? The diagnosis and treatment of the setting and system she was living and being diagnosed in is more of a challenge than the diagnosis of the patient!’^38^

Shocked at the turn of events relayed by the case report, the ostensive concern of the respondent is for more contextual information about the patient and her situation which explains the delay in diagnosing a disease (Wilson's disease) that usually responds to treatment with copper chelating agents: treatment which could have halted and reversed the appalling decline in the woman's health. The correspondent, a professor of family medicine, demands to know how a human life could have been allowed to fall apart without someone responding to her plight earlier. The letter shares a response to the report that references wider realms of human significance - biographical disruption and cultural and personal meanings – than are easily accommodated by narrow construals of medical problems for the purposes of diagnosis.^39^ The correspondent registers dissatisfaction with the narrow framing of the report in purely neurological terms, his focus being on the woman and her social and personal circumstances over and above the disease and diagnosis.

Archie Cochrane's account of a man he attended at Elsterhorst, a German prisoner of war camp in Saxony in 1943, also challenges case construction from two different perspectives:

‘The Germans dumped a young Soviet prisoner in my ward late one night. … He had obvious gross bilateral cavitation and a severe pleural rub. I thought the latter was the cause of the pain and the screaming. I had no morphia, just aspirin, which had no effect. I felt desperate. I knew very little Russian then and there was no one in the ward who did. I finally instinctively sat down on the bed and took him in my arms, and the screaming stopped almost at once. He died peacefully in my arms a few hours later. It was not the pleurisy that caused the screaming but loneliness. It was a wonderful education about the care of the dying. I was ashamed of my misdiagnosis and kept the story secret.’^40^

The case appeared in a memoir published many years after these events took place and it has since entered the medical literature as an example of good palliative care.^41^ Initially Cochrane believed it was pain that accounted for the man's ‘screaming’ for which he had no treatment, pain due to ‘a severe pleural rub’ caused by tuberculous inflammation of the linings of the lung grating on each other during inspiration. But he retracts the facticity of this sequence and replaces it with another: that it was loneliness, a different mental state (not pain) about which the man had been screaming.^42^ This claim has the effect of de-medicalizing the concern away from pathology, medical causation and inadequate analgesia towards a focus on the man's existential situation: on how prolonged physical closeness could meet an inner need for accompaniment and still the dying soldier, allowing him to pass peacefully away. Within the account there is a tension between a focus on the person who is dying and the attempt to pinpoint the medical cause of his behaviour, set against the tumultuous background of the physician's own emotions, thoughts and attempts to muster an adequate response to him:

‘I put him in my room as he was moribund and screaming as I did not want to wake the ward. I examined him. … I had no morphia…. I felt desperate. I knew very little Russian then…. I finally instinctively sat down on the bed and took him in my arms…. I was ashamed of my misdiagnosis and kept the story secret.’

Cochrane's growing sense of desperation dominates the account and it is through his emotional reactions as physician-narrator that we learn of the urgency of the situation. Such inner aspects of the hurly burly of clinical work are usually edited out of case reports; and if they do creep in it is wonder, awe and astonishment that become apparent in response to clinical appearances^43^ rather than shame and desperation. It is rare to sense the raw desperation, shame and tenderness seen here; instinctive gestures amounting almost to a caress lasting hours being conspicuous by their absence in case reports. In the period in which these events unfolded, taking a patient in one's arms as a physician might have been considered improper; perhaps publishing the case outside the confines of a peer reviewed journal allowed Cochrane to bring his feelings to the fore as part of an effort to resolve what had taken place between himself and the dying soldier. Its power and pungency stem from recounting the ‘parade of present moments’ that make up clinical experience with its ‘reflective pauses’ and ‘takings-stock’^44^ recalled from a vantage point decades distant from the events themselves. These cases highlight the role of hindsight in developing perspectives that generate meaning which goes beyond narrow diagnostic formulations.

## 4 Narratives of diagnosing

Clinical case reporting generally privileges a medical perspective, which muffles patients' voices, calls into question the subjective aspects of illness experiences, and the reliability of patient testimony. In modern case reports:

‘the patient's concern is termed a complaint; his or her voice … [and] information is scant. Use of the passive voice eliminates agency: for example, ‘the patient was treated with...’ and ‘the patient was noted on physical examination to have...’… [which] makes invisible the persons making observations and decisions and performing procedures. …. [S]pecific linguistic markers encode scepticism about patients' accounts, with phrases such as ‘the patient reports’ or ‘the patient claims’ denoting these portions of the case as subjective perceptions which may or may not be factual. The perspective dominating the account is that of the doctor, for the case history is not the patient's story. It is the doctor's highly structured rendering of certain aspects of the patient's experience.’^45^

A modern medical textbook claims that ‘taking a patient's history and giving advice in clinical consultation is an exchange of narratives',^46^ yet over the past century-and-a-half, patients' points of view expressed in case reports have progressively diminished. The historian of medicine Jonathan Gillis finds the patient's history to have become ‘a construct and production of the clinical encounter, rather than a simple expression of the patient's narrative’, such that there are now two histories in play in case reporting: ‘a superficial, overt, story presented by the patient … and a deep, covert, and “true” history revealed by …the physician.’^47^ The latter makes sensible how the diagnostic process will attempt to identify the *p* chosen for the patient and case at hand.

The primary means by which modern cases engage with patients' accounts is by excerpting snippets of testimony on their felt experiences and articulating them with third-party formulations derived from the differing modalities of clinical assessment - conversation, observation, physical examination, and medical investigation. The resulting formulations generate the data from which the case is assembled through a process of representation which positions symptoms as the originary point of the report^48^:

‘A 39-year-old right-handed community nurse presented to us on the neurology ward in April, 2005, after a telephone call from her work colleagues. They expressed concern that she had appeared subdued before starting the afternoon shift and that, when asked, she could not recall her home address. She had apparently carried out her morning duties as normal and could not understand her colleagues’ concerns. However, she agreed to be reviewed. The nurse's symptoms had begun 48 h previously, when she woke with a bitemporal headache, similar in character to previous episodes of migraine. Her vision then “flipped 180°” so that all images appeared inverted. She was able to crawl back to bed and on waking 6 h later, her vision had returned to normal and the headache had subsided. There were no further symptoms, and she continued engagements over the weekend as planned. She had experienced a similar episode while at work 12 years previously. Her vision had “flipped” again, occurring in combination with a migraine.’^49^

In foregrounding disturbances of mood and memory the entrée to this case registers differing views on their significance, including the nurse's own evaluation - that little about her situation seemed amiss to her – which contrasts with the viewpoints of her colleagues who arranged hospital assessment. Hints of such differences fade as the focus moves to very particular disturbances of the nurse's memory and visual perception, situated through glances *forwards* to the weekend following their onset, *backwards* to register a similar experience twelve years earlier, then forwards again to the *present*, to consider the nurse's current situation. This spatiotemporal positioning of the patient's experiences within a history frames the account, drawing further information from medical notes, third-party reports of laboratory and imaging studies, and physician memory.^50^

A case which focuses on diagnosis starts life during the clinical consultation in attempts to identify facts about the patient germane to the possible causes of illness. Empirical studies show that the vast majority of patients attending hospital clinics can be diagnosed by taking account of the medical history alone, results of clinical and laboratory examinations proving less frequently contributory to formulating a diagnosis.^51^ Frank Davidoff, a former editor of *the Annals of Internal Medicine* sees the published case as a post-consultation representation: ‘a bird's eye, after-the-fact version of the diagnostic process.’^52^ Thus through retrospection, developments unfolding in the future time of the case influence the way the clinical events and happenings which eventuate are presented in a report. Temporal change and stasis – a simultaneous grasping of beginning and end along the lines Louis Mink conceived of for historical narrative – play a role in the encapsulation of spatiotemporal clinical patterns:

‘… in the configurational comprehension of a story which one has followed, the end is connected with the promise of the beginning as well as the beginning with the promise of the end… To comprehend temporal succession means to think of it in both directions at once, and then time is no longer the river which bears us along but the river in aerial view, upstream and downstream in a single survey.’^53^

The future is immanent in the way the author selects and discounts, highlights and de-emphasises clinical features of interest: ‘Data about the patient [are] trimmed of irrelevant historical, physical, and laboratory findings’^54^ argues Jerome Kassirer, a former editor of the *New England Journal of Medicine*, in order to focus on inter-connectable symptoms and signs that conform to ‘textbook examples (paradigms, prototypical cases)’.^55^ Experiences deemed incidental to such patterns may be treated as non-contributory even though they may have been crucial to the patient.^56^ Case reports not only configure through constructing a historical narrative, they also present a version of the medical encounter (a ‘single survey’ in Mink's terms) that displays a neat and rational picture of the diagnostic process, excluding or downplaying information important to the patient and masking disorderly features of clinical phenomena.^57^

It is not just that diagnostic reports benefit from hindsight: cases unfold as re-formulations of clinical appearances that commence at time T1 under a description D1 and are supplanted by subsequent descriptions D2 at T2, which significantly were not available at T1. One way a case report reaches a decision about which *p* is relevant, and explains that choice, is by shifting the description under which a symptom or clinical phenomenon is first encountered - often in snippets of the patient's own words - to an account constructed in quite different terms, emerging through eventuation and from the results of clinical assessment and investigation. This is shown by the way the nurse's visual experiences come to be represented and understood.

Explanation of her ‘180° reversal of vertical vision metamorphopsia’^58^ is developed through attempts to retrace pathological processes and possible causal mechanisms that can support a plausible aetiology (see Crasnow, and Currie and Sterelny in this issue): magnetic resonance imaging (MRI) of the nurse's brain revealed a recent and older lesion in the thalamus, a midline area known to be associated with visual image orientation. Hypotheses that could account for recurrent damage to the area are not explicitly developed in the case report, but almost certainly will have been considered by the patient's doctors. Only the most likely causes, severely diminished or blocked cerebral blood flow, gain attention because an ultrasound assessment of the nurse's vasculature revealed a hitherto undiagnosed congenital connection between the left and right cardiac atria known to cause turbulent blood flow and to predispose to thrombus formation. Although no trace of a thrombus was seen on the scan, the supposition is that at some point after a clot formed it broke away from its point of origin in the heart and embolized - travelled up the arteries of the neck to her brain – to injure the thalamus as visualized on MRI scan. Through a combination of observation, hypothesis and reasoning, a causal account is developed of how the nurse's cerebral pathology (D2) eventuated.^59^

The nurse's symptoms (D1) might have been explained by different thalamic pathologies such as infective, traumatic or haemorrhagic damage. But traces of such insults - a history of fever in relation to cerebral abscess, signs of trauma on X-ray or of altered blood clotting revealed by in vitro testing – do not feature in this particular account, which is teleologically organised around thalamic stroke, not around other possible pathologies. “The Case of the Forgotten Address” is an end-based account of how evidence to support a particular diagnosis was put together rather than of how a final diagnosis was discriminated from other likely causes. Because a certain circulatory hypothesis explained the findings in the nurse's case - an inference grounded in part on considerations of relative likelihoods not discussed in the report - the authors infer the truth of that hypothesis.

Links in this chain of causation carry differing epistemic status. Because there is no direct evidence of intra-cardiac clot formation and its subsequent movement to the brain, the causal mechanism remains an inferred one based on likelihood. The report exhibits no scrutiny of the similarity between the two clinical episodes experienced by the nurse; their time course is not compared feature by feature in terms of associated symptoms such as nausea, unsteadiness and disorientation. The similarity between the episodes remains impressionistic: no mention is made in the earlier one of loss of memory (a defining characteristic of the ‘case’ according to the report's title), altered cognitive ability or of the nurse's apparent affective indifference, which are marked features of the recent episode (‘she could not understand her colleagues’ concerns').^60^ The match rests on the nurse's experience, memory, and on her capacity to make a judgement of similitude across a long time interval, which the report does not corroborate, for example, in her answers to further questions. The authors accept an identity relation between the two episodes holds; and that each belongs to the same type of syndrome, which suggests that any explanatory hypothesis – the choice of ‘which *p*?’ - will need to account for two separate events of the same kind.

The diagnosis is also grounded in matching the nurse's medical situation to those described in other case reports of strokes of this sort^61^ which Forrester (after Kuhn) identified as ‘reasoning and working in shared examples’.^62^ Such cases have highlighted similar visual and cognitive disturbances manifest as behaviour described as ‘robot-like’, evidenced in the nurse continuing to work despite the onset of alarming visual symptoms. Her headache, anterograde amnesia and a low score on formal cognitive testing (all of which are detailed later in the report) are taken to be features sufficiently similar to those set out in earlier cases as to corroborate the diagnosis. Thalamic stroke caused by a heart defect underpins the rationale of treatment: to make the nurse's blood less coaguable and dissolve the current clot (if any of it remains), preventing further intra-cardiac clot formation. At the time the case was published - nine months after the nurse's admission to hospital - her clinical state had vastly improved: she had gone back to work and had not experienced any recurrence of the symptoms.^63^

The report is a highly edited account of clinical practices which translate the nurse's symptoms – including those of the earlier period - into a series of pathological processes. Grounded in comparisons with clinical patterns in previous case reports, technologies of looking such as ultrasound and magnetic resonance scanning, and in what is known of the circulatory physiology and pathology of a common cardiac anomaly, the report also draws on what is understood of symptoms associated with damage to the thalamus.^64^ The case is assembled from a mass of evidence arising from the patient herself; her colleagues' testimony, and from examination and investigation of her body. A succession of biological processes is posited which supports an account of how the syndrome could have arisen twice, the cardiac lesion being the ‘smoking gun’, the evidential sign of a common cause^65^ that in narrative terms enables structurally and functionally distinct events to be tied together in one story. The nurse's voice - the initiatory stimulus of the case – is clearly heard at the start of the account, but fades as the authors' technical re-description in anatomical and physiological terms gets under way and as the narrative moves towards a diagnosis.

No such process of re-description takes place in the case of injury to the workman's boot because no explanation of the man's symptoms appears in the account. Without a somatic injury the diagnosis cannot be likened to apprehending how the incidents of a story belong together in Mink's sense,^66^ because the end of the plot is not connected with the promise of the beginning, since no physical process links the uninjured foot to severe pain. If, as Arthur Danto argues, stories require a beginning, middle and an end, and the ‘explanation then consists in filling in the middle between end-points of a change’^67^ the point of interest in this case lies precisely in its narrative gap, the missing explanatory middle.

Despite this ellipsis the case of the uninjured foot remains an end-organised narrative, one arranged around irresolution rather than resolution, which works by replacing one puzzle with another. Yet its significance goes beyond the staging of a clinical riddle solely for amusement; its publication is justified by an untheorized but important observation that answers the generic question to which all non-fraudulent clinical cases respond affirmatively: ‘Is it possible for such-and-such to occur?’^68^ The answer calls attention to the knowledge-bearing nature of this case and alerts readers to the entanglement of physical and psychological processes in experiences of pain.

Although the clinical history is posited to be the ‘basic experiential unit’^69^ of medical thinking, the discourse of the case report does not offer unproblematic access to medical phenomena. The accounts of the uninjured foot and the dying soldier synchronously align knowledge with surprise, which subverts the view that the medical problem can be taken for granted at the level of description. They show that *p* is subject to radical reformulation at the level of observation; and it is apparent from the cases that knowledge of a diagnosis does not predict all the important details of the experience of illness or the trajectory of a condition in an individual.^70^

## 5 Conclusion: the narrative format

Why do case reports published in today's general medical journals articulate clinical understanding through cadences of arousal and resolution embodied in narratives? The diagnostic criteria of diseases do not subsist in narrative form; even conditions featuring a pronounced spatiotemporal dimension such as multiple sclerosis – which requires evidence of pathological processes at different moments in time in different parts of the central nervous system - are formulated as conditional statements concerning the presence or absence of attributes or functions, not as narratives^71^; and although information in clinical records may feature storied scenes recounted by patients and carers, the overall shape of these records is much more like a sequential listing of happenings and findings than the elaborate, second order configurations of narrative considered here.

A sense of audience looms large in the way case reports bring information from different sources together, releasing it along linear, jumpy and looping time-lines, foreshadowing and foreclosing on possible futures (see Beatty this issue). Such stagings engage readers' imaginations, playing on their expectations whilst also challenging their powers of pattern recognition and reasoning.^72^ The narrative shape of the case is not confined to published accounts; ethnographers have commented on how clinical presentations in hospitals are frequently staged as suspenseful dramas, ‘surgeons artfully creating stories about patients and their conditions … akin to mysteries or cliff-hangers, sometimes morality tales of success and failure.’^73^ Although audience hardly featured in Forrester's account of “thinking in cases” it turns out to be a prominent influence on the manner in which information is orgnanised and disclosed,^74^ reasoned with, and made memorable in contemporary clinical case reports.^75^

The selection of clinical communiqués considered here has encompassed a range of reporting practices, displaying forms that indicate the potential of the case report genre to make sense of widely differing clinical situations. The task is met by creating a history, ‘a narrative that brings together diverse past facts into a causal account’^76^ concerning how a particular *p* came about, one which grants authorial viewpoint latitude in the way its features are presented and encapsulated.^77^ Encapsulations inflect epistemological gradients of uncertainty that wax at the start of an account and wane as data are generated which stimulate hypotheses and various sorts of inferences. Although case-based diagnostic reasoning moves from recounted experiences, bodily effects and traces, to antecedent causes and mechanisms, reports are bound up with hindsight, with perspective-taking from the future and the retrofitting of information, which suggests that the process of diagnosis, and so the choice of which *p* is relevant, appears rational and unifying.^78^

The brevity of reports considered here leads them to feature a high ratio of essential to incidental details, while the balance between forward clinical reasoning in the context of uncertainty, and retrospective understanding in the context of hindsight, has a critical bearing on how a clinical case is seen to unfold.^79^ By deploying devices that shape and channel attention^80^ plots are generated that rely on readers assuming a future is in place waiting to be revealed. But how precisely this future will play out, and how it will be manifested, is a decision for the authors. Reports draw on all the modalities of clinical assessment - conversation, observation, physical examination and investigation – in which the boundaries between the particulars of illness and generic conceptions of disease, and between the person who is ill and the depersonified patient, may shift and become indistinct.^81^ Amidst hypotheses and mechanisms invoked to explain what eventuates, the literary and narrative machineries of the case interweave their medical subject matters, through which the fortunes of individuals come into view. Thus case reporting is pervious to narrative discourses such as the sentimental tale,^82^ eccentric biography,^83^ and suspense and detective fiction that are capable of telling how the trajectories of a life, and of an illness, intersect diagnostically.

## Footnotes

1 Forrester, John, ‘If *p*, then what? Thinking in cases’, *History of the Human Sciences* 1996; 9(3) 1–25. See also his recently collected papers on cases: Forrester, John, *Thinking in Cases*, Cambridge, Polity Press, 2017.

2 In their written form such pathways include not only the case history according to Gianna Pomata, but also the aphorism, dialogue, essay and medical recipe, all of which she counts as an ‘epistemic genre’ in medicine. See Pomata, Gianna, ‘The Medical Case Narrative: Distant reading of an Epistemic Genre.’ *Literature and Medicine*, 2014; 32(1): pp. 1-23. See also: Morgan, Mary S, ‘Case Studies: One Observation or Many? Justification or Discovery?’ *Philosophy of Science* 2012; 79(5): 667-77.

3 Berman P, Horton R, **‘**Case Reports in The Lancet – a new narrative.’ *Lancet, 2015; 385: 1277*.

4 Berman, P, Horton R, ‘Case Reports’ *Lancet, 2015*.

5 Bignall J, Horton R, ‘Learning from stories—The Lancet's Case Reports.’ *Lancet* 1995; 346: 1246. Although the language of case reports tends to efface their origins in human and healthcare relationships, the authors of a study of evidence in practice point to the importance of informal relations and communications in the development of clinical understanding: ‘Coffee-room chat may impact on evidence-based practice at least as much as all those guidelines that deluge GPs… [and]… we need to understand how and why that is…’. Gabbay, John, Andreé le May. ‘Mindlines: making sense of evidence in practice’, *BJGP* 2016; 66:402-3 at 402.

6 See Vandenbroucke, Jan P, ‘In Defense of Case Reports and Case Series.’ *Ann Intern Med*. 2001; 134:330–334. See also: Carey, John C, ‘Significance of Case Reports in the Advancement of Medical Scientific Knowledge’, *American Journal of Medical Genetics* Part A 2006; 140A:2131–2134; van der Wall, E.E., ‘Case history: more than a beauty case!’ *Netherlands Heart Journal* 2008; 16 (7/8): 235–6.

7 By syndrome is meant a repeatedly observed configuration of clinical features believed to reflect a pathological mechanism or class of phenomena not fully explained, such as autism, anorexia-bulimia, or post-traumatic stress disorder. See: Aronowitz, Robert A, ‘When Do Symptoms Become a Disease?’ *Ann Intern Med* 2001;134:803–808. See Morgan's discussion of colligation in this issue.

8 Aronson, Jeffrey K, Manfred Hauben, ‘Anecdotes that provide definitive evidence’, *BMJ* 2006;333:1267–1269.

9 Smalheiser, Neil R, Weixiang Shao, Philip S. Yu, ‘Nuggets: findings shared in multiple clinical case reports.’ *J Med Lib Assoc* 2015; 103(4): 171–6 at 171. The revaluation of case reports has taken place both in general medical journals and in journals founded solely to publish cases. See: Akers, Katherine, ‘New journals for publishing medical case reports’, *J Med Libr Assoc* 2016; 104(2): 146–9; van der Wall, E.E., ‘Case history: more than a beauty case!’ *Netherlands Heart Journal* 2008; 16 (7/8): 235–6.

10 Forrester 1996 p 14.

11 Forrester, John, ‘On Kuhn's Case: Psychoanalysis and the Paradigm.’ *Critical Inquiry* 2007; 23: 782–819 at p 810.

12 DeBakey, Lois, Selma Debakey, ‘The case report. I. Guidelines for preparation’, *International Journal of Cardiology* 1983;4:357–64, p 358.

13 Glasziou, Paul, Iain Chalmers, Michael Rawlins, Peter McCulloch, ‘When are randomised trials unnecessary? Picking signal from noise.’ *BMJ* 2007;334; 349–351 doi:http://dx.doi.org/10.1136/bmj.39070.527986.68.

14 Prince,Gerald, *Narratology: The Form and Function of Narrative*, Berlin: Mouton, 1982; pp. 4, 145; Prince, Gerald, *A Dictionary of Narratology*, Lincoln and London: University of Nebraska Press, 1987; p. 59.

15 The factors mentioned as possibly bearing on the outcome of this procedure, have pertained to the nature, geometrical and material properties of the misplaced objects in the nose – tissue paper, snippets of cloth, plasticine, small toys, food items, polystyrene foam, magnets, pen parts, crayon pieces and living creatures such as lavae and worms – omit reference to possible child-centred factors such as gender, age, personality, mental health, or to the relationship with the parent and parental competence in performing the manoeuvre.

16 Fischer, Jonathan, ‘Nasal Foreign Bodies.’ *Medscape* August 2015, http://emedicine.medscape.com/article/763767-overview?pa=G%2FU0EkyrUiTnPaNEsuFEgu99QFCcoSRbI4SjH0mts2H4oLW3vAjCIjVOB5LkABisLCEJNCrbkqLWYvqLrhntWA%3D%3D#a7 [retrieved 15 Nov 2016].

17 It is not clear whether this account, written by a group of physician-scientists (one of whom several years later published a systematic review of case series featuring this manoeuvre) arises from personal witness; the fact that it is written in the third person suggests it may be a composite of several cases of this complaint and its treatment, an illustration of a typical example of it.

18 Pooled outcomes in one systematic review have not found these factors to predict success. See: Cook, Stephanie, Burton, Martin, Glasiou, Paul, ‘Efficacy and safety of the “mother's kiss” technique: a systematic review of case reports and case series.’ *Can Med Assoc Journal* 2012; 184(17): E904–E912. doi:http://dx.doi.org/10.1503/cmaj.111864.

19 The importance of child cooperation is signified in the name that has come to be associated with the procedure. See Guazzo, Eugene, ‘Removal of Foreign Bodies from the Nose.’ *NEJM* 1985;312:725, Botma, Marissa, Bader, Rixana, Kuba, Haytham, ‘A parent's kiss: evaluating an unusual method of removing nasal foreign bodies in children.’ *J Laryngol & Otology* 2000;114: 598–600. In the Cook *et al* study some of the case series list a care giver rather than a parent as the blower; success rates between series vary from 100% to 49%, and rates between smooth and irregular nasal objects are not distinguishable but numbers are small, the number of cases in series ranging from 100 to 2. See: Cook, Stephanie, Burton, Martin, Glasiou, Paul, ‘Efficacy and safety of the “mother's kiss” technique’ 2012.

20 Norman, Richard, *On Humanism,* Routledge 2004; 152–3. Norman argues that some narratives gain their power from configuring a particular set of events readers recognise as a ‘paradigmatic particularity’. p 153.

21 Turner, Henry S, ‘Lessons from Literature for the Historian of Science (and Vice Versa).’ *Isis* 2010; 101: 578–589 at 585.

22 Sturdy, Steve, ‘Knowing Cases: Biomedicine in Edinburgh, 1887–1920’, *Social Studies of Science* 2007; 37: 659–689 at p 676.

23 See Rousseau, George, ‘Discourses of Literature and Medicine: Theory and Practice (1).’ In *Enlightenment Borders*, 2–25. Manchester: Manchester University Press, 1991 at p 10. See also Clark, William. ‘Narratology and the History of Science.’ *Stud Hist Phil Sci* 1995 vol 26.1, 1–71 at 56.

24 Tougaw, Jason Daniel, *Strange Cases – The Medical Case History and the British Novel*. NY & London: Routledge 2006; p 49.

25 Hunter, Kathryn Montgomery, *Doctors' Stories: The Narrative Structure of Medical Knowledge,* Princeton: Princeton University Press, 1991; p 66.

26 Herzog, Todd, ‘Crime Stories: Criminal, Society, and the Modernist Case History’. *Representations* 2002; 80: 34–61 at p 34 quoting from Doyle, Arthur Conan, *The Hound of the Baskervilles*, in *The Illustrated Sherlock Holmes Treasury*. New York 1976;527–632 at p 616. See also Schum, David A, *Thoughts About a Science of Evidence*. London: University College London 2005; pp 1-70. https://www.ucl.ac.uk/jdi/research/evidence-network/docs/Science.doc accessed 20/02/2017.

27 Sussex, Lucy, *Women Writers and Detectives in Nineteenth-Century Crime Fiction: The Mothers of the Mystery Genre*. Basingstoke: Palgrave Macmillan 2010; pp. 10, 18–24.

28 Rick Rylance notes that some clinical features of nineteenth-century cases are treated in the pages of medical journals as ‘bizarre… grotesque … freakish’ and ‘gothic’. Rylance, Rick,‘The Theatre and the Granary: Observations on Nineteenth-Century Medical Narratives’, *Literature and Medicine* 2006; 25: 255–276 p 269.

29 Punter, David, *Gothic Pathologies: The Text, the Body and the Law*. London: Palgrave Macmillan 1998; p 6.

30 Levine, Carole, *The Serious Pleasures of Suspense: Victorian Realism and Narrative Doubt*. Virginia: University of Virginia Press 2003; p 3.

31 Levine, *Serious Pleasures* p 74.

32 Fisher JP, Hassan DT, O'Connor N, *BMJ* 1995; 310:70. (Unusually this case image is untitled).

33 Rylance ‘The Theatre and the Granary’ p 270.

34 Some of these questions arose in discussions of an earlier draft of this paper with Michael Flexer.

35 Robert Aronowitz makes a similar point when he draws attention to the tension between medical conceptualisations and the person who is the patient: ‘At the clinical level, we need to recognize and accommodate the essential continuity between persons who have symptoms that have been given a name and disease-like status and persons whose suffering remains unnamed and unrecognized’. See Aronowitz ‘When Do Symptoms Become a Disease?’ p 808.

36 Epstein, Julia, ‘Historiography, Diagnisis and Poetics’, *Literature and Medicine* 1992; 11(1): pp. 23–44 at 33.

37 Davendralingam N, I Davagnanam, A Djamshidian, A Lees. ‘Progressive dysphagia, dysarthria, dystonia, and tremor.’ *BMJ* 2010;340:c1213 doi:http://dx.doi.org/10.1136/bmj.c1213 accessed 10/07/2016.

38 Glaser, Anthony N, ‘Why so long?’ http://www.bmj.com/rapid-response/2011/11/02/why-so-long accessed 10/07/2016.

39 As Annemarie Mol puts it, whatever medical problems pertaining to the body one is faced with ‘they are situated elsewhere: in one's life.’ Mol, Annemarie, *The Body Multiple: Ontology in Medical Practice*. North Carolina: Duke University Press 2002; p 127.

40 Cochrane, Archie, L, (with Max Blythe), *One man's medicine*, London: British Medical Journal (Memoir Club) 1989; p 82.

41 Wiffen, P, ‘The Cochrane collaboration: pain, palliative and supportive care’, *Palliative Medicine* 2003; 17: 75–77.

42 Alternatively, it is possible that the soldier was in severe pain all along and that the pain abated or became much more bearable as a result of this act of recognition, which allowed him to pass away peacefully.

43 For a fuller treatment of this case see Hurwitz, Brian, ‘What Archie Cochrane Learnt from a single case’. *Lancet* 2017; 389: 594-5. On the affects associated with curious medical cases see Kennedy, Meegan, *Revising the Clinic*, Ohio: Ohio State University Press 2010, especially pp 30–53.

44 Freeman, Mark, *Hindsight. The Promise and Peril of Looking Backward*. Oxford: Oxford University Press 2010; p 4. A hindsight view favours enactment in and through the past-tense which Roland Barthes called ‘the cornerstone of Narration’. Almost invariably this is the ‘Narrative mode’ of medical case reports: ‘The verb implicitly belongs with a causal chain … [a]llowing as it does an ambiguity between temporality and causality …. Its function is no longer that of a tense … [but] to abstract, from the depth and multiplicity of experiences … a logical link …’. See: Barthes, Roland, *Writing Degree Zero*, translated by Annette Lavers and Colin Smith, London: Jonathan Cape, 1967; 30–31.

45 Kenny, Nuala P., Brenda L Beagan, ‘The Patient as Text: A Challenge for Problem-Base Learning.’ *Medical Education* 2004; 38: 1071–79.

46 Jenicek, Milos, *A Primer on Clinical Experience in Medicine*. Boca Raton, London, New York: CRC Press 2014; p 236.

47 Gillis, Jonathan, ‘The History of the Patient History since 1850’, *Bull Hist Med* 2006; 80(3):490–512 at 512. Kathryn Montgomery makes similar points but narratologically: ‘[t]he two stories have different authors, different narrative modes, different themes. The patient's story … is not simply “raw data.” The patient has constructed it in an effort to make sense of life-altering events.’ Hunter, Kathryn Montgomery, *Doctors' Stories: The Narrative Structure of Medical Knowledge* Princeton: Princeton University Press 1991; p 62. See also Epstein, Julia, ‘Historiography, Diagnosis, and Poetics’, *Literature and Medicine* 1992;11: 23–44.

48 Oliver Sacks puts this point clearly: ‘Typically… my case histories start with an encounter, a letter, a knock on the door – it is the patients’ description of their experience that stimulates the more general explanation.’ Sacks, Oliver, *The Mind's Eye*. London: Picador 2010; Preface p x.

49 Samarasekera S, Dorman P, ‘The case of the forgotten address’, *Lancet* 2006; 367:1290.

50 See Warner, John Harley, ‘The Aesthetic Grounding of Modern Medicine’, *Bulletin of the History of Medicine* 2013; 88 1–47. Warner, John Harley, ‘The Uses of Patient Records by Historians – Patterns, Possibilities and Perplexities’, *Health and History* 1999; 1: 101–11. Sturdy, Steve. ‘Knowing Cases: Biomedicine in Edinburgh, 1887–1920’, *Social Studies of Science* 2007; 37: 659–689.

51 Hampton, John R, MJ Harrison, J R Mitchell, J S Pritchard, C Seymour, ‘Relative Contributions of History-Taking, Physical Examination, and Laboratory Investigation to Diagnosis and Management of Medical Outpatients’, *BMJ* 1975; 2(5969): 486–89; Peterson MC, J H Holbrook JH, D Von Hales, et al., ‘Contributions of this history, physical examination, and laboratory investigation in making medical diagnoses’, *West J Med* 1992;156:163–5.

52 Davidoff, Frank, ‘Teaching files, textbook examples – the case of the classic case.’ Paper presented at the Annual Meeting of the American Educational Research Association, Atlanta, Ga., April 12–16, 1993 http://acpinternist.acponline.org/archives/1995/04/textbk.htm?print=true accessed 04/05/16, reprinted in Davidoff, Frank, *Who has seen a blood sugar?* Philadelphia: American College of Physicians 1996; 5–6.

53 Mink, Louis 0, ‘History and Fiction as Modes of Comprehension.’ *New Literary History* 1970;1(3):541–558 p 555.

54 Kassirer, Jerome, ‘Clinical Problem Solving – A New Feature in the Journal.’ *NEJM* 1992; 326(1):60–61.

55 Davidoff ‘Teaching files’ p1.

56 Wade, Derek T., Peter W Halligan, ‘Do biomedical models of illness make for good healthcare systems?’ *BMJ* 2004; 329: 1398–1401.

57 Part of what goes into making case reports appear so ‘neat’ and ‘rational’ is the privileged knowledge hindsight has of what transpires in a case, all of which may not be known by the attending clinicians grappling with formulation of a diagnosis, the sort of historical knowledge to which Arthur Danto refers when he writes that: ‘The whole truth concerning an event can only be known after the event itself has taken place.’ Danto, Arthur C, *Narration and Knowledge*, Columbia: Columbia University Press 1985 (revised edition of Analytical Philosophy of History 1968), p151. See also White, Hayden, ‘Historiography and Historiophoty’, *The American Historical Review*, 1988; 93(5): 1193–1199.

58 The medical term metamorphopsia refers to visual images which are distorted in shape, size or position.

59 Samarasekera, ‘The case of the forgotten address’ 1290.

60 Third person testimony was available from the second episode but the nurse's first episode may have been unwitnessed; and if it caused alterations in memory it may not have been possible to gain further corroboration about the relationship between the two episodes.

61 The authors cite an early description of visual inversion from 1868 in which there was no clear neurological localisation and two papers on thalamic strokes: Carrera, L E, Michel P, Bogousslavasky, J, ‘Anteromedian, central and posterolateral infarcts of the thalamus’, *Stroke* 2004; 35: 2826–31, and Schmahmann, J, ‘Vascular syndromes of the thalamus’, *Stroke* 2003; 34: 2264–78. On visual inversion syndromes and their associated phenomena see: Aysun, Unal, Cila, Aysenur, Saygi Serap, ‘Reversal of vision metamorphopsia: A manifestation of focal seizure due to cortical dysplasia.’ *Archives of Neurology* 1998;55(10):1362–8; and Solms, Mark, Karen Kaplan-Solms, Michael Saling, Percy Miller, ‘Inverted Vision After Frontal Lobe Disease’, *Cortex* 1988; 24(4):499–509.

62 Forrester, ‘On Kuhn's Case’ 2007 at p 813.

63 To the extent that the outcome of this medical assessment is apparent in the report up to nine months after the onset of the second episode, the efficacy of anticoagulation cannot be inferred simply from the lack of a recurrence in the nurse's case, especially in the light of the fact that she improved without this treatment after the earlier episode, which did not recur for some twelve years.

64 Steve Sturdy calls this a process of ‘triangulation’ drawing on Susan Leigh Star's *Regions of the Mind: Brain Research and the Quest for Scientific Certainty* Stanford, CA: Stanford University Press, 1989; 96–117. See also: Sturdy, Steve. ‘Knowing Cases: Biomedicine in Edinburgh, 1887–1920.’ *Social Studies of Science* 2007; 37: 659–689.

65 Cleland, Carol E, ‘Methodological and Epistemic Differences between Historical Science and Experimental Science.’ *Philosophy of Science* 2002; 69 (3): pp. 447–451 http://www.jstor.org/stable/10.1086/342455.

66 Mink, Louis 0, ‘History and Fiction as Modes of Comprehension.’ *New Literary History*, 1970; 1(3): 541–558 at p 555.

67 Danto *Narration and Knowledge* 1985; p 233.

68 Millar, Janice M, Harry M Whitaker,‘The Right Hemisphere's Contribution to Language: A Review of the Evidence for Brain Damaged Subjects.’ In Sydney J Segalowitz (ed) *Language, Functions and Brain Organization*. London: Academic Press 1983, pp 87–111 at p102 (emphasis in original text).

69 Jacyna, Stephen, *Lost Words: Narratives of Language and the Brain, 1825–1926* Princeton, NJ and Oxford: Princeton University Press 2000; p24.

70 See Ankeny, Rachel A, ‘The Overlooked Role of Cases in Causal Attribution in Medicine,’ *Philosophy of Science* 2014; 81(5): 999–1011.

71 Polman, Chris H, Stephen C Reingold, Brenda Banwell, Michel Clanet, Jeffrey A Cohen, Massimo Filippi et al., ‘Diagnostic criteria for multiple sclerosis: 2010 Revisions to the McDonald criteria’, *Ann Neurol*. 2011; 69(2): 292–302.

72 Kathryn Montgomery Hunter, *Doctors' Stories: The Narrative Structure of Medical Knowledge* Princeton: Princeton University Press, 1991; Hurwitz, Brian, ‘Narrative and the practice of medicine’, *Lancet* 2000; 356: 2086–2089.

73 Atkinson, Paul, *Medical Talk and Medical Work: The Liturgy of the Clinic,* London: Sage, 1995; p. 4. See also Atkinson, Paul, ‘Narrative Turn or Blind Alley?’ *Qualitative Health Research* 1997; 7 (3) pp 325–44. For the role of narrative in teaching case based thinking see Good, Byron J, Mary-Jo Delvecchio Good, ‘“Fiction” and “Historicity” in Doctors’ Stories', in Cheryl Mattingly and Linda C. Garro (eds.), *Narrative and the Cultural Construction of Illness and Healing,* Berkley: University of California Press 2000; pp 50–69; Good, Byron J, *Medicine, rationality and experience: An anthropological perspective,* Cambridge: Cambridge University Press, 1994.

74 The titles of some modern case reports are clearly oriented to garnering readers as in Ogundare O, Jumma O, Turnbull D, Woywodt A, ‘Searching for a needle in the Haystacks’, *Lancet* 2009;374:850. Dahms, Kirsten, Yulia Sharkova, Peter Heitland, Sabine Pankuweit, Juergen R Schaefer, ‘Cobalt intoxication diagnosed with the help of Dr House.’ *Lancet* 2014; 383: 574 DOI:http://dx.doi.org/10.1016/S0140-6736(14)60037-4.

75 Gilbert Ryle maintained that: ‘Being good at recalling is … being good at presenting. It is a narrative skill’. Ryle, Gilbert, *The Concept of Mind,* London: Hutchinson, 1949; p. 272.

76 Forrester ‘On Kuhn's Case’ at p 800.

77 Brooks, Peter, *Reading for the Plot: Design and Intention in Narrative*, Cambridge Mass: Harvard University Press, 1992; p 23.

78 Freeman discusses this in terms of an ‘illusory appearance of order and coherence’ which hindsight as a form of ‘narrative reflection’ can generate. See Freeman *Hindsight* pp 23–4.

79 A systematic sampling of case reports at 10-year intervals from 1947 to 2016 covering *The New England Journal of Medicine*, *The Lancet* and the *British Medical Journal* finds that the more condensed and summarized the form that medical case reports take, the more likely they are to assume a narrative form (unpublished findings of a study by Flexer, Michael and Brian Hurwitz, 2016).

80 Pomata, Gianna, ‘Sharing Cases: The *Observationes* in Early Modern Medicine’, *Early Science and Medicine* 2010; 15(3):193-236.

81 See Foucault's formulation of ‘a case which at one and the same time constitutes an object for a branch of knowledge… and… the individual as he may be described, judged measured, compared with others, in his individuality;’ in Foucault, Michel, *Discipline and Punish*: *The Birth of the Prison*, Translated by Alan Sheridan. London: Allen Lane, 1977; p 191.

82 See for example, Chandler, James, ‘On the Face of the Case: Conrad, Lord Jim, and the Sentimental Novel’, *Critical Inquiry* 2007;33 (4): pp. 837–864; and Hurwitz, Brian, ‘Urban Observation and Sentiment in James Parkinson's Essay on the Shaking Palsy’, *Literature and Medicine* 2014; *32: 1, 74–104.* DOI:http://dx.doi.org/10.1353/lm.2014.0002.

83 See for example, Hoare, J, ‘Extraordinary narrative’, *Lancet* 1832;20–1; and Gregory J, ‘Eccentric Biography and the Victorians.’ *Biography* 2007; 30: 342–76.

